# Solvent-Free Esterification of Carboxylic Acids Using Supported Iron Oxide Nanoparticles as an Efficient and Recoverable Catalyst

**DOI:** 10.3390/ma9070557

**Published:** 2016-07-12

**Authors:** Fatemeh Rajabi, Mohammad Abdollahi, Rafael Luque

**Affiliations:** 1 Department of Science, Payame Noor University, P.O. Box 19395-4697, Tehran 19569, Iran; mohammadabdollahichem@gmail.com; 2 Departamento de Química Orgánica, Universidad de Córdoba, Campus de Rabanales, Edificio Marie Curie (C-3), Ctra Nnal IV-A, km 396, Cordoba 14014, Spain; q62alsor@uco.es

**Keywords:** supported iron oxide nanoparticles, esterification, carboxylic acid

## Abstract

Supported iron oxide nanoparticles on mesoporous materials (FeNP@SBA-15) have been successfully utilized in the esterification of a variety carboxylic acids including aromatic, aliphatic, and long-chain carboxylic acids under convenient reaction conditions. The supported catalyst could be easily recovered after reaction completion and reused several times without any loss in activity after up to 10 runs.

## 1. Introduction

Esters play a significant role in daily living and the chemical industry. The reaction of carboxylic acids with alcohols to form esters is among the mildest and most efficient of organic transformations, largely a consequence of the high accessibility and stability of reactants. High ester usage in the synthesis of drugs, fine chemicals, pharmaceuticals, solvents and plasticizers as intermediates makes these substrates one of the most important types of compounds in organic chemistry [1].

In this context, some protocols for the synthesis of esters are well-known, including Fisher esterifications [2] and methylation reactions [3] of carboxylic acids. Due to the wide synthetic and biological applications of esters, a number of reagents such as ortho esters [4], *N*,*N*-dimethylformamide dialkyl acetals [5], triazene derivatives [6] and *O*-dialkyl isoureas [7] have been reported for the esterification of various aromatic/aliphatic carboxylic acids.

**Scheme 1:**
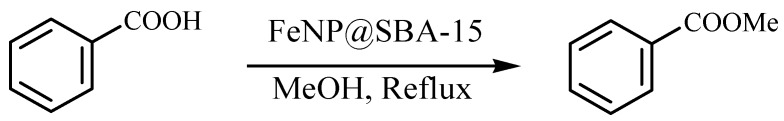
Esterification of benzoic acid with FeNP supported on SBA-15.

The reaction of carboxylic acids and alcohols in the absence of catalysts is very slow and requires a long time for the reaction to reach equilibrium. To accelerate reaction rates, a number of catalysts have been reported inthe literature. These include classical solid acids such as ion exchange resins [8,9,10], zeolites [11,12], super acids [13,14], heteropolyacids [15,16,17,18] and supported chlorides [19]. Metal oxides such as CaO and MgO [20], metal-layered hydroxides [21,22], and efficient enzymatic catalysts [23,24] have also been employed in esterification reactions. However, many of these methods suffer from inherent drawbacks such as the need for expensive or harmful materials as reagents and catalysts, the formation of undesired side products, highly acidic conditions, the use of hazardous and toxic solvents, high reaction temperatures, low yield of products and prolonged reaction times. A need to develop an improved catalytic system for the synthesis of esters in terms of operational simplicity and economic viability is of utmost importance. Herein, a nanomaterial based on supported iron oxide nanoparticles on SBA-15 (FeNP@SBA-15) has been utilized as an efficient catalyst for a mild esterification of various carboxylic acids to their corresponding esters (Scheme 1). The combination of iron nanoparticles and the mesoporous structure of the material showed excellent synergistic effects on the enhancement of activity and stability of the catalyst. Apart from a high activity, the successful recycling of this catalytic system allows a more economic and environmentally friendly process which is of special advantage for large-scale preparations and industrial applications.

## 2. Results and Discussion

The catalytic performance of supported iron oxide nanoparticles has been previously reported by our research group [25,26,27]. In continuation of our previous study on the application of FeNP@SBA-15 as a recoverable catalyst [26], we found that the esterification of carboxylic acids in the presence of FeNP@SBA-15 as an effective catalyst has not been investigated yet. Hence, we decided to investigate the catalytic effect of FeNP@SBA-15 as a promoter system on the rate and efficiency of esterification of carboxylic acids. The material FeNP@SBA-15 has been previously described and characterized by a series of techniques including Inductively coupled plasma/Mass spectrometry (ICP/MS), X-ray diffraction (XRD), Scanning electron microscopy (SEM), Transmission electron microscopy (TEM) and X-ray photoelectron spectroscopy (XPS) [27].

**Figure 1:**
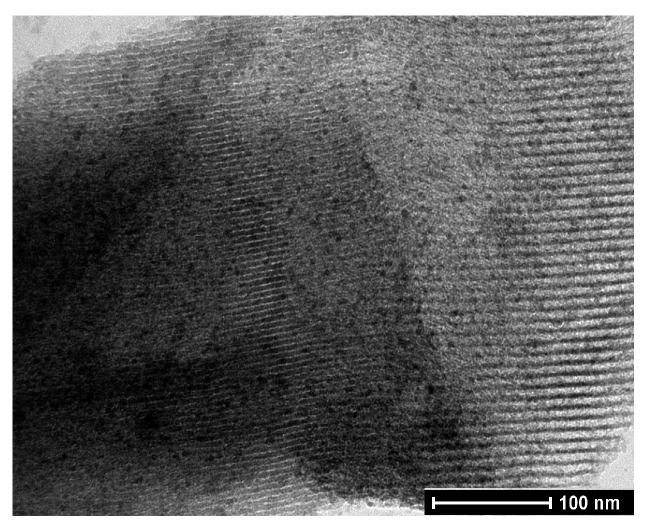
TEM micrograph of FeNP@SBA-15 material.

**Figure 2:**
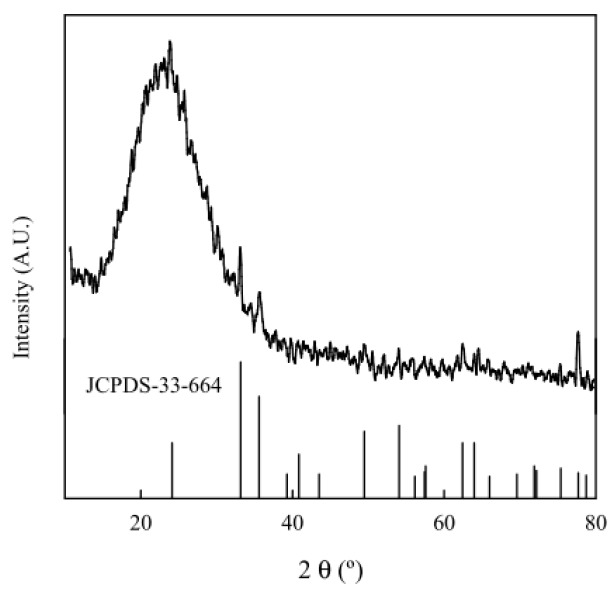
XRD pattern of FeNP@SBA-15. Bottom lines correspond to the JCPDS 39-0664 card of hematite phase Fe_2_O_3_.

**Figure 3:**
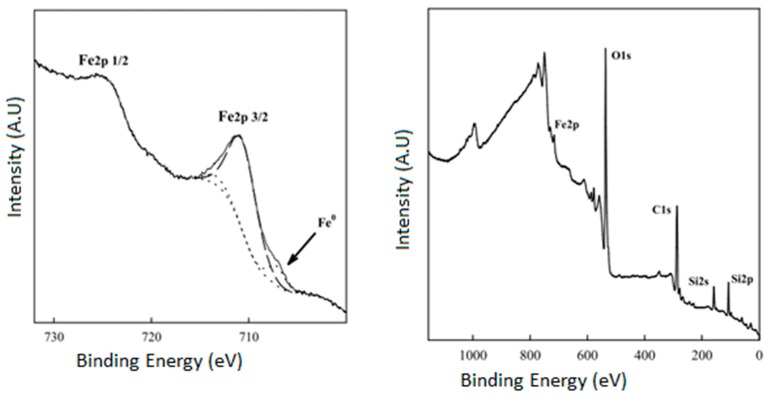
XPS spectra of Fe2p (**left panel**) and survey (**right panel**) of FeNP@SBA-15.

TEM images of the catalyst indicated that the iron oxide nanoparticle sizes were in the 5–7 nm range, with an excellent homogeneous dispersion of the iron oxide nanoparticles on the support (Figure 1). Fe species in the synthesized materials as measured by ICP/MS were found to be around 0.5–0.6 wt. %, with average iron oxide nanoparticle sizes in the 5–8 nm range. XRD of the materials confirmed the presence of the hematite phase (Fe_2_O_3_, JCPDS card 39-0664) for FeNP@SBA-15 (Figure 2), which was also confirmed by XPS measurements (typical Fe^3+^ bands at BE 714 eV(Fe2p_3/2_) and 725 eV (Fe2p_1/2_), Figure 3), with only a very minor contribution (<2%) of zerovalent Fe.

The influence of FeNP@SBA-15 in the esterification of benzoic acid with methanol was firstly investigated (Table 1). Blank runs indicated that the esterification of benzoic acid with methanol in the absence of FeNP@SBA-15 did not take place at 60 °C (Table 1, Entry 1). A simple addition of 0.3 mol. % FeNP@SBA-15 as catalyst to the mixture of benzoic acid (1 mmol) and methanol (2 mmol) already provided a low yield of methylbenzoate (10%) at room temperature (Table 1, Entry 2). The optimization of the reaction conditions eventually led to quantitative yields of the corresponding ester (Table 1, Entry 4). Eventually, the reaction of benzoic acid (1 mmol) with methanol (2 mmol) in the presence of 0.1 mol. % FeNP@SBA-15 under methanol reflux was selected as the optimum condition for the esterification reaction due to the high efficiency and short reaction times (typically, 6 h, Table 1, Entry 8).

The efficiency and scope of the present protocol was further extended to a broad range of aromatic and aliphatic carboxylic acids containing electron-donating or -withdrawing groups with methanol and ethanol as esterifying reagents under optimized reaction conditions. As shown in Table 2, most carboxylic acids underwent esterification to afford the corresponding esters in excellent yields (88% to 99%). The introduction of substituents often changes the activity of the aromatic ring but changing the aromatic substitution from an electron-donating group to an electron-withdrawing group did not significantly influence yields to products as clearly indicated in Table 2. Our system exhibited an almost analogous efficiency towards both activated and non-activated aromatic carboxylic acids (Table 2, Entries 2 and 6). The α,β-unsaturated carboxylic acids were also efficiently esterified to the corresponding esters without any observable reaction at the double bond (Table 2, Entries 16–18). The reaction of aliphatic carboxylic acids with alcohols in this work did not show obvious differences and the corresponding esters were also obtained in high yields (Table 2, Entries 10–11). Only sterically hindered carboxylic acids were less reactive (in comparison with unhindered) in the catalytic system under optimum reaction conditions (Table 2, Entry 8). Interestingly, a biomass-derived platform chemical such as succinic acid (a C4 diacid) could be efficiently esterified to dimethyl succinate in high yield (Table 2, Entry 12).

The solventless reaction was also performed with solid phase alcohols. As example, the reaction between benzoic acid and 4-chlorobenzyl alcohol (1:1) under optimized conditions for 12 h could provide an isolated ester yield of 55%. The presence of the organic ligand grafted on the SBA-15 surface did not seem to have any effect on the catalytic activity in the systems, with a negligible esterification activity observed for aminopropyl-functionalized SBA-15 (in the absence of Fe_2_O_3_ NPs).

The esterification reaction of benzoic acid with methanol catalyzed by several different catalysts reported in the literature has been summarized in Table 3. As can be seen, the catalytic performance of FeNP@SBA-15 was remarkably improved as compared to data reported in the literature in terms of catalytic activity and mol. % of used catalyst.

**Scheme 2:**
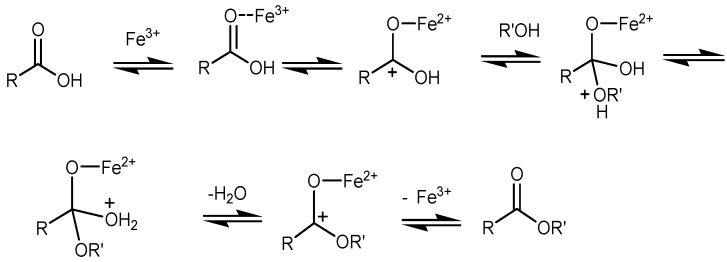
Lewis acid-catalyzed esterfication mechanism.

A proposed reaction mechanism for the esterification is shown in Scheme 2 and Fe species are coordinated to the carbonyl oxygen, followed by intermediate generation and alcohol addition to generate the observed ester products in high yields.

**Figure 4:**
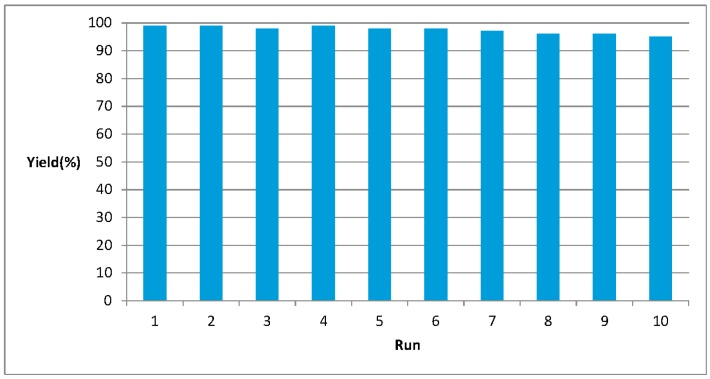
Recycling of the supported FeNP and the yield of isolated methylbenzoate in 10 subsequent runs. Reaction conditions: 2 mmol benzoic acid, 4 mmol MeOH, 7 mg FeNP@SBA-15 (0.2 mol. %) at reflux conditions for 6 h.

After reaction completion, the possibility of reusing supported FeNP catalyst was determined. The catalyst was easily separated from the reaction mixture through filtration, washed with ethyl acetate to remove residual product and reused in a subsequent reaction. As an example, the reaction of benzoic acid and methanol in the presence of FeNP@SBA-15 afforded methylbenzoate in quantitative yields even after 10 successive runs under optimized reaction conditions, with an average yield of 97%, supporting the stability and reusability of the catalytic system (Figure 4). Furthermore, XPS spectra of the catalyst recorded after the several runs show that the Fe^3+^ species are mostly present in the catalyst.

## 3. Materials and Methods

### 3.1. General Information

Unless otherwise stated, all reagents and chemicals in this study were used as received and were not further purified (Sigma-Aldrich Chemie GmbH, Taufkirchen, Germany). Melting point was recorded on a RY-1 microscopic melting apparatus (Hangzhou Chincan Trading Co., Shanghai, China) and uncorrected. ^1^H-NMR and ^13^C-NMR spectra were respectively recorded on 500 MHz and 125 MHz by using a Bruker Avance 500 spectrometer (Bruker BioSpin GmbH, Rheinstetten, Germany). Metal content in the materials was determined using inductively coupled plasma (ICP) in a Philips PU 70000 sequential spectrometer (Philips, Almelo, The Netherlands) equipped with an Echelle monochromator (0.0075 nm resolution). Samples were digested in HNO_3_ and subsequently analyzed by ICP. Nitrogen adsorption measurements were carried out at 77 K using an ASAP 2000 volumetric adsorption analyzer from Micromeritics (Micromeritics, Norcross, GA, USA). The samples were outgassed for 24 h at 100 °C under vacuum (10^−2^ Pa) and subsequently analyzed. Powder X-ray diffraction patterns were recorded on a Bruker-AXS diffractometer using a Cu Kα radiation (λ = 1.5409 Å). XPS measurements were performed in an ultra-high vacuum (UHV) multipurpose surface analysis system (Specs™) operating at pressures <10^−10^ mbar using a conventional X-ray source (XR-50, Specs, Mg-Kα, 1253.6 eV) in a “stop-and-go” mode to reduce potential damage due to sample irradiation. The survey and detailed O and Si high-resolution spectra (pass energy 25 and 10 eV, step size 1 and 0.1 eV, respectively) were recorded at room temperature with a Phoibos 150-MCD energy analyzer (SPECS GmbH, Berlin, Germany). Powdered samples were deposited on a sample holder using double-sided adhesive tape and subsequently evacuated under vacuum (<10^−6^ Torr) overnight. Eventually, the sample holder containing the degassed sample was transferred to the analysis chamber for XPS studies.

### 3.2. Preparation of Aminopropyl-Functionalized SBA-15 Materials (SBA-15-NH_2_)

Co-condensed amino-SBA-15 silicas were synthesized according to the procedure described by Wang et al. [33]. Aminopropyl-functionalized SBA-15 materials (denoted as SBA-15-NH_2_) were prepared by a one-pot synthesis method. Pluronic 123 (4 g) was dissolved in 125 g of 2.0 M HCl solution at room temperature. After TEOS was added, the resultant solution was equilibrated at 40 °C for prehydrolysis, and then APTES was slowly added into the solution. The molar composition of the mixture was 0.9 TEOS: 0.1 APTES: 6.1 HCl: 0.017 P123:165 H_2_O. The resulting mixture was stirred at 40 °C for 20 h and then reacted at 90 °C under static condition for 24 h. The solid product was recovered by filtration and dried at room temperature overnight. The template was removed from the material by refluxing in excess ethanol for 24 h. Finally, the material was filtered, washed several times with water and ethanol, and dried at 50 °C.

### 3.3. Preparation of Supported Iron Oxide Nanoparticles (FeNP@SBA-15) 

Salicylaldehyde (2 mmol, 0.244 g) was added to excess absolute MeOH, to which Aminopropyl-functionalized SBA-15 materials (2.35 g, loading of NH_2_ group is 0.85 mmol/g) were then added. The solution became yellow due to imine formation. After 6 h, Fe(NO)_3_·9H_2_O, (1 mmol), was added to the solution, and the mixture was stirred for a further 24 h to allow the new ligands to complex the iron and a red brown color was observed. The final product was washed with MeOH and water until the washings were colorless. Further drying of the solid product was carried out in an oven at 80 °C for 8 h. 

### 3.4. General Reaction Procedure

In a typical reaction, 0.005 mmol of supported FeNP (0.5 mol. %) was added to a mixture of carboxylic acid precursor (5 mmol) and excess ROH (molar ratio 1:2) under reflux conditions for 6 h. The reaction progress was monitored by using thin-layer chromatography (TLC), after completion of the reaction; the catalyst was separated from the mixture through filtration and then washed with portions of 20 mL ethyl acetate and heated at 70 °C prior to its reuse in the next reaction. The combined filtrate and ethyl acetate washings were then washed with water and the organic layer separated and dried over magnesium sulfate. The product was obtained after removal of the solvent.

## 4. Conclusions

A promising, efficient and green approach for the synthesis of various esters via reaction between carboxylic acids and alcohols in the presence of catalytic amounts of low-loaded iron oxide nanoparticles on SBA-15 materials under solvent-free conditions was successfully performed. The supported iron oxide nanocatalyst exhibited a remarkable stability under these conditions and could be easily removed from the reaction mixture by simple filtration and reused 10 times without any significant loss in activity. The versatility, convenient operation, and cost-effectiveness of this approach, in addition to the high yields, make it highly attractive both in laboratory research and potentially for scaling up.

## Figures and Tables

**Table 1:** Screening of reaction conditions in the esterification of benzoic acid with methanol ^a^.

Entry	FeNP (mol. %)	Time (h)	T (°C)	Yield (%) ^b^
1	-	10	60	-
2	0.3	10	r.t.	10
3	0.3	10	40	22
4	0.3	10	reflux	99
5	0.2	10	reflux	99
6	0.1	10	reflux	99
7	0.07	10	reflux	48
**8**	**0.1**	**6**	**reflux**	**99**
9	0.1	4	reflux	91

^a^ All reactions were carried out with 1 mmol of benzoic acid and 2 mmol MeOH; ^b^ Isolated yield.

**Table 2:** Esterification of carboxylic acid derivatives using supported iron oxide nanoparticles ^a^.

Entry	Carboxylic Acid	Product (Ester)	Alcohol	Yield (%) ^b^
1	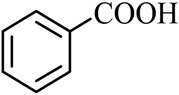	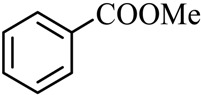	CH_3_OH	99
2	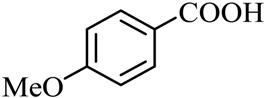	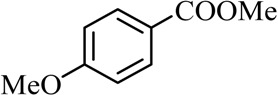	CH_3_OH	98
3	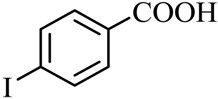	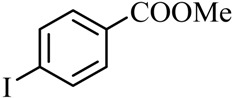	CH_3_OH	97
4	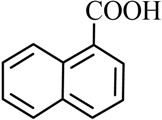	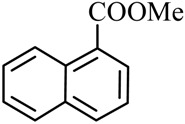	CH_3_OH	96
5	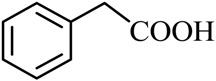	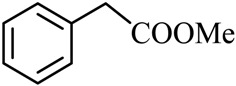	CH_3_OH	90
6	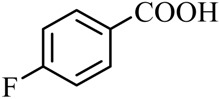	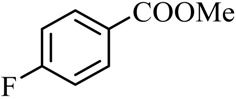	CH_3_OH	94
7	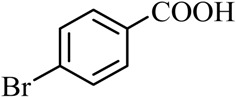	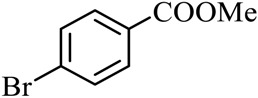	CH_3_OH	95
8	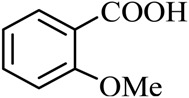	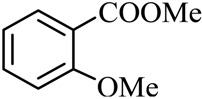	CH_3_OH	88
9	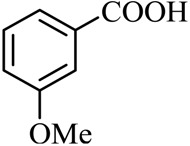	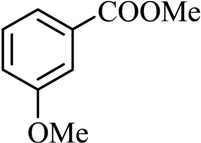	CH_3_OH	90
10	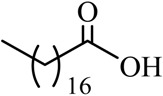	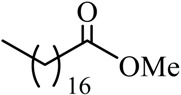	CH_3_OH	93
11	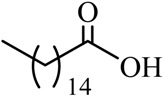	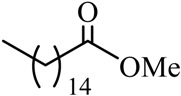	CH_3_OH	95
12	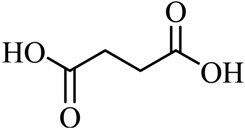	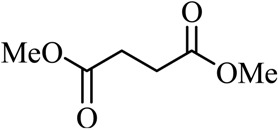	CH_3_OH	90
13	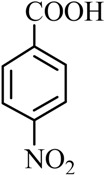	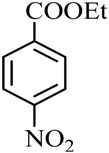	CH_3_CH_2_OH	90
14	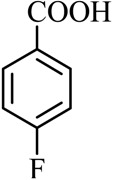	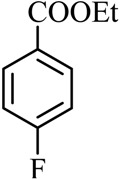	CH_3_CH_2_OH	92
15	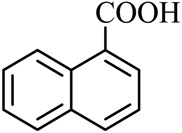	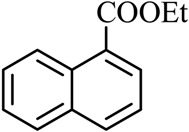	CH_3_CH_2_OH	94
16	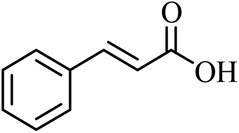	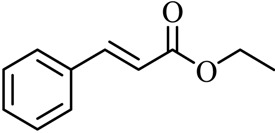	CH_3_CH_2_OH	95
17	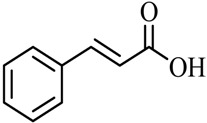	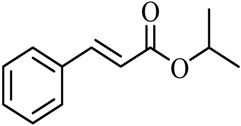	(CH_3_)_2_CHOH	95
18	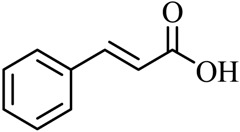	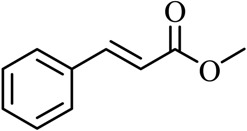	CH_3_OH	98

^a^ All reactions were carried out with the molar ratio of substrate/ROH (1:2) in the presence of supported iron oxide nanoparticles (0.1 mol. %) at reflux conditions for 6 h; ^b^ Isolated yields.

**Table 3:** Comparison of various systems in the esterification of benzoic acid by methanol.

Entry	Catalyst	Mol (%)	T (°C)	Acid/Methanol Molar Ratio	Time (h)	Yield(%) ^a^	Ref.
1	FeNP@SBA-15	0.1	reflux	1:2	6	99	^b^
2	CoNP@SBA-15	0.5	reflux	1:2	12	98	[28]
3	[C_3_SO_3_Hmim]HSO_4_	0.3	95	1:3	2	98	[29]
4	Ionic liquids based on benzothiazoliumcations	5	120	1:4	8	97.9	[30]
5	Pd/C	0.5 gr	60	1:excess amount	4	90	[31]
6	HClO_4_-SiO_2_	1	100	1:1	3	96	[32]

^a^ Isolated yield; ^b^ Present work.
